# High efficiency transformation of *Brassica oleracea* var. *botrytis* plants by *Rhizobium rhizogenes*

**DOI:** 10.1186/s13568-018-0656-6

**Published:** 2018-08-06

**Authors:** Tomasz Kowalczyk, Aneta Gerszberg, Paulina Durańska, Róża Biłas, Katarzyna Hnatuszko-Konka

**Affiliations:** Department of Genetics, Plant Molecular Biology and Biotechnology, Faculty of Biology and Environmental Protection, University of Lodz, Banacha 12/16, 90-237 Lodz, Poland

**Keywords:** *Brassica oleracea* var. *botrytis*, Cauliflower, Regeneration, Transformation, *Rhizobium rhizogenes*

## Abstract

*Brassica oleracea* var. *botrytis*, a very popular crop grown for its edible inflorescence, is bred only as a mutated annual cultivar and does not naturally occur in environment. Since cauliflower is still described as the most troublesome of all the *B. oleracea* vegetables regarding transformation processes, it is fully justified to focus on the improvement of tools for its genetic modifications. Here, we present a successful protocol for genetic transformation of cauliflower employing the process of agroinfection. The primary analysis of in vitro response of five cultivars allowed us to have chosen Pionier as the most promising cultivar; in consequence the Pionier was transformed via *Rhizobium*-mediated techniques in order to evaluate both, *R. radiobacter* (EHA 105, LBA 4404) and *R. rhizogenes* (ATCC 18534, A4) species. However, the latter system turned out to be more effective and, the A4 strain, in particular (72% transformation efficiency, 55% confirmed by GUS assay). That shows a promising technical advance especially when compared to the results of previous literature reports (e.g. 8.7% reported efficiency using *R. rhizogenes*). The transgenic cauliflower was obtained from hairy roots via organogenic callus induction. The potential transformants were analysed at the genomic and proteomic levels and their transgenic character was fully confirmed.

**Electronic supplementary material:**

The online version of this article (10.1186/s13568-018-0656-6) contains supplementary material, which is available to authorized users.

## Introduction

*Brassica oleracea*, a species commonly known for its polymorphism, comprises a number of varieties of significant importance for human consumption. This opulent species includes, among others, cabbage, broccoli, brussels sprouts and cauliflower, and because of its nutritional importance is a subject of both conventional and biotechnological breeding and modifications (Vinterhalter et al. 2007). Moreover, some of the cole crops (e.g. broccoli and cauliflower) were proved to reduce the risk of cancer in several organs (Spini and Kerr 2006). For these reasons, research on genetic engineering of the *Brassicaceae* family has aroused huge interest in recent years, especially since a quite good background of repeatable regeneration protocols became available. As it can be seen from the literature data, the prerequisite for genetic engineering—a plant regeneration system for in vitro propagation—has been established for many *Brassica* vegetables, e.g. reports by Gerszberg et al. 2015; Kumar and Srivastava 2016; Munshi et al. 2007; Pavlović et al. 2010; Ravanfar et al. 2014; Shahid et al. 2010. Genetic transformation of the cole crops was achieved by direct DNA uptake and by the *Rhizobium*-mediated technique (Bhalla and Singh 2008; Chakrabarty et al. 2002; Nugent et al. 2006; Radchuk et al. 2002). However, those closely related botanical varieties obviously display substantial differences in the response to regeneration and transformation approaches (Pavlović et al. 2010; Sretenović-Rajićić et al. 2006). Thus, a successful protocol for one genotype does not mean a fruitful transformation for another.

Of all the *B. oleracea* vegetables, cauliflower seems to be the least amenable to genetic transformation. It is particularly troublesome due to its economic importance. *B. oleracea* var. *botrytis* is grown in nearly 100 countries worldwide and suffers from the lack of disease and pest tolerance, and in consequence from decreasing quality and crop yield. Cauliflower is known to contain high amounts of vitamins C, K, A and B9, fiber and flavonoids, which results in its antioxidant and anti-inflammatory properties and anticancer activities (Metwali and Al-Maghrabi 2012). Similarly to other *Brassica* plants, cauliflower was regenerated under in vitro conditions via different pathways and from different explants: direct and indirect somatic embryogenesis from hypocotyl and leaf explants (Leroy et al. 2000; Siong et al. 2011) or organogenic shoot regeneration from hypocotyl, cotyledon or root explants (Pavlović et al. 2010). Like in the case of other cole crops, organogenesis is the most popular method for the cauliflower tissue culture (Shahid et al. 2010). Requirements for the cauliflower tissue culture are simple, but strongly diverse. The literature data suggest that the hypocotyl of cauliflower seems to be an explant of choice for the studies on regeneration, pointing at its amenability to in vitro handling (Brown and Wang 2004).

To date, only a few traits have been introduced into cauliflower via genetic engineering, although its transformation has been achieved by several techniques. To the best of our knowledge, there are still no reports on a stable genetic modification of cauliflower via the biolistic approach; only transient expression studies were reported by Brown and Wang (2004). Instead, this variety was modified by both direct DNA uptake (electroporation, PEG-mediated modification) and via the *Rhizobium radiobacter* and *Rhizobium rhizogenes*-mediated transformation (Chikkala et al. 2008; Kumar Ray et al. 2012; Radchuk et al. 2002; Theriappan and Gupta 2014). However, despite those successful approaches, cauliflower is considered to be quite recalcitrant to genetic engineering and displays cultivar-dependent response to such modifications.

In this paper we focus on refining the transformation process to genetically improve *B. oleracea* var. *botrytis* plants. We present the complete procedure of the production of transgenic cauliflower plants, starting from a brief evaluation of the regeneration potential (the screening prephase) of different cultivars and culture media, then presenting different approaches to genetic engineering of cauliflower, and ending with the analysis of potentially transgenic cauliflower plants. The process of the preliminary screening prephase to identify the most promising target cultivar has been briefly presented.

## Materials and methods

### Plant material

#### Screening prephase—methods

Five randomly selected cultivars of *B. oleracea* var. *botrytis* were employed for primary screening: Di Sicilia Violetto, Pionier, Poranek, Rober, Snowball X. Since we have drawn on the experience and results from the available literature, we decided to test the regeneration potential of cotyledons and hypocotyls (Brown and Wang 2004; Pavlović et al. 2010; Qamar et al. 2014). Also the medium composition was established based on the available reports. The regeneration potential of both hypocotyls and cotyledons was tested on five medium variants: pure basal Murashige and Skoog medium (MS) (1962) supplemented with 1% sucrose and 0.8% agar as a control (***A***) and four combinations of the MS medium supplemented with different concentrations of growth regulators (***B*** − MS + 1 mg/L BAP, ***C*** − MS + 0.5 mg/L BAP + 0.1 mg/L NAA, ***D*** − MS + 1 mg/L BAP + 0.1 mg/L NAA, ***E*** − MS + 2 mg/L BAP + 0.1 mg/L NAA)*;

*NAA naphthaleneacetic acid, *BAP* 6-benzylaminopurine.

Surface sterilized seeds were spread on a ½ MS medium supplemented with 1% sucrose and 0.8% agar to achieve 100 seedlings for each cultivar (the detailed procedure of sterilization is presented in the “Explants for transformation” subsection). Finally, 100 cotyledon (one cotyledon from one seedling to make the numbers of explants equal) and 100 hypocotyl explants from 10-day-old seedlings were bred on each medium variant and shifted regularly to a fresh medium every 2 weeks.

The regenerated multiplied shoots of all cultivars were moved to root forming media: ***A***^***r***^ − MS, ***B***^***r***^ − MS + 0.5 mg/L NAA, ***C***^***r***^ − MS + 1 mg/L NAA, ***D***^***r***^ − MS + 1.5 mg/L NAA; all supplemented with 1% sucrose and 0.8% agar.

#### Explants for transformation

Seeds of the Pionier (target) cultivar (PNOS PlantiCo Ltd; Zielonki, Poland) were sterilized in 70% ethanol for 1 min and rinsed three times with sterile-distilled water for 3 min each time. Next, they were treated with 50% ACE solution (commercial bleach containing 5% sodium hypochlorite) for 5 min followed by another two rinses in distilled water. The sterilized seeds were spread on a ½ MS germination medium (Murashige and Skoog 1962) supplemented with 1% sucrose and 0.8% agar, pH 5.6–5.8 and kept under 16 h/8 h (light/dark) photoperiod in 25 °C. 10-day-old seedlings were used for two transformation approaches:

- 200 hypocotyls were processed for the *R. radiobacter*-mediated transformation;
- 200 derooted seedlings were processed for the *R. rhizogenes*-mediated transformation.

### Binary vector

All the *Rhizobium* strains used for the plant material transformation were harboring the pCAMBIA 1305.2 binary vector (from the Commonwealth Scientific and Industrial Research Organization, CSIRO, Australia http://www.cambia.org/) (GenBank: AF354046.1). The plasmid was modified in our department by Dr. Piotr Łuchniak and carries the Elastin-Like Polypeptides (*ELP*) sequence fused to the native *β*-glucuronidase gene (*gusPlus* sequence available as a part of pCAMBIA 1305.2, protein ID AAK29427.1) (Żelazowski 2005). A circular plasmid map was generated using the SnapGene software (from GSL Biotech; available at http://www.snapgene.com, Chicago, USA) and is presented as Additional file 1: Fig. S1.

### Bacterial strains

Source information for each strain is presented below:

- *R. rhizogenes* ATCC 18534 (*Agrobacterium rhizogenes*) (https://www.lgcstandards-atcc.org/products/all/15834.aspx?geo_country=pl);
- *R. rhizogenes* A4 (*Agrobacterium rhizogenes*) (https://www.lgcstandards-atcc.org/Global/Products/2/A/4/0/43057.aspx);
- *R. radiobacter* EHA105 (*Agrobacterium tumefaciens*) (https://www.lifescience-market.com/p/eha105-agrobacterium-strain-p-63289.html);
- *R. radiobacter* LBA4404 (*Agrobacterium tumefaciens*) https://www.thermofisher.com/order/catalog/product/18313015).

A single colony of each bacteria strain was picked-up and transferred to 5 mL of the YEP medium with 50 mg/L of kanamycin and cultured in the dark at 28 °C on a rotary shaker at 120 rpm for 24 h. Afterwards, 100 mL of the YEP medium with 50 mg/L of kanamycin was inoculated with 2 mL of the bacteria cultures and cultured in the dark at 28 °C on a rotary shaker at 100 rpm until transformation.

### Plant transformation

#### LBA 4404 and EHA 105 *Rhizobium radiobacter* strains

Since the Pionier hypocotyls have demonstrated the greatest regeneration potential, they were chosen as the target explants for genetic engineering. The explants were transferred to the regeneration medium: MS + 1 mg/L 2,4-d (2,4-dichlorophenoxyacetic acid). The pre-culture was kept for 48 h at 25 °C with a 16 h/8 h photoperiod. Then, the explants were transferred to a bacterial solution (a dilutions 1:20 of OD_600_ absorbance of 0.5) for 30 min (100 hypocotyls to the LBA 4404 strain and 100 hypocotyls to EHA 105, both harboring the modified pCAMBIA 1305.2 vector). 50 μM acetosyringone was used as a transformation enhancer. The explants were dried on sterile filter paper to remove the excess of bacteria. Next, the explants were transferred to the co-cultivation medium (MS + 2 mg/L BAP + 0.5 mg/L IAA) (Chakrabarty et al. 2002). The co-culture was kept in the dark for 2 days at 25 °C. After 2 days of co-culture, the explants were rinsed in the MS medium with 500 mg/L of cefotaxime, dried on sterile filter paper and transferred to the selective medium I° (MS + 2 mg/L BAP + 0.5 mg/L IAA + 500 mg/L cefotaxime). The culture was kept for 7 days (delay period) at 25 °C with a 16 h/8 h photoperiod. The explants were transferred to the selective medium II°, which contained both antibiotics (a double selective medium: variant *E* + 500 mg/L cefotaxime + 50 mg/L hygromycin B). The culture was kept for 14 days at 25 °C with a 16 h/8 h photoperiod and moved to a fresh medium II° every 2 weeks (the winning regeneration medium from the screening prephase: *E *− MS + 2 mg/L BAP + 0.1 mg/L NAA). The explants were planned to be transfer to the fresh medium until adequate shoots appeared and grew.

#### ATCC 15834 and A4 *Rhizobium rhizogenes* strains

Hairy roots on plant material were obtained as follows. 10-day-old seedlings without roots were taken for transformation (100 derooted seedlings for the ATCC 15834 strain and 100 for A4). All bacteria strains were grown on liquid YEP medium for 24 h at 28 °C before transformation. Afterwards, the bacteria cultures were centrifuged (4000 rpm, 15 min) and the pellet was resuspended and diluted in liquid MS medium supplemented with 1 mg/L of 2,4-d to OD_600_ = 0.3. The seedling hypocotyl zone was immersed for 1 min in the bacteria harbouring modified pCAMBIA 1305.2 vector, suspended in the MS medium. The plant material was dried on sterile filter paper to remove the excess of bacteria and placed upside-down on a Petri dish with the MS medium solidified with 0.8% agar. The inoculated plantlets were incubated under a 16 h/8 h (light/dark) photoperiod at 25 °C until hairy roots appeared (3–4 weeks). Then, each individual transgenic root was cut off from the seedling hypocotyl and placed on a Petri dish with the MS medium with 500 mg/L of cefotaxime and 50 mg/L of hygromycin B. The roots were incubated in the dark at 25 °C. After 7 days, well growing root clones were tested for GUS activity with histochemical assay.

### Transgenic plant regeneration

#### Regeneration from hypocotyls

To obtain shoots for transgenic plant regeneration the hypocotyls subjected to agroinfection were regularly transferred to a fresh selective medium, variant *E*. As early as only after 10 days first regenerating shoots could have been observed. After 2 months the shoots were excised and moved to the rooting medium (rooting variant *A*).

#### Regeneration from roots

To obtain callus tissue for transgenic plant regeneration, selected hairy roots were transferred to the MS medium with 5 mg/L BAP and NAA, 3% sucrose, solidified with 0.8% agar, partly based on Puddephat et al. (2001). Petri dishes with the roots were incubated in the dark at 24 °C and the proliferating tissue was transferred to a new medium every 3 weeks. New developing shoots were excised and transferred, first to the regeneration medium variant *E,* and then to the MS medium with 1% sucrose and incubated until rooting (rooting medium variant *A*). The whole transgenic plants were cultured under sterile conditions.

#### PCR analysis of the potential transformants

Plant genomic DNA was isolated from leaves by the method described in Doyle and Doyle (1990). PCR primers were designed to amplify a 488 bp fragment of *gusPlus* (forward, 5′-ttgtctatgtcaatggtgagc-3′, and reverse, 5′-cactttgatctggtagagatacg-3′). The PCR amplification procedure was as follows: 95 °C—4 min, 54 °C—1 min, 72 °C—2 min, 30 cycles of 94 °C—45 s, 54 °C—30, 72 °C—1 min, ending with a final extension at 72 °C—5 min. The PCR products were separated in 1% agarose gel.

### Western blot analysis of the transgenic plants

Western blot analysis was performed according to the method described by Sambrook et al. (1989). To analyze the transgenic nature of the obtained cauliflower plants at the proteomic level, the total extractable protein content was isolated by the modified method by Hurkman and Tanaka (1986). Next, the cauliflower protein extracts were separated by SDS-PAGE (Laemmli 1970). Electrophoresis was conducted for 2 h at 180 V (Mini-Protean 3 apparatus, Bio-Rad, Hercules, USA). Wet electrotransfer was conducted in the Bio-Rad device (Mini Trans-Blot Cell, Bio-Rad, Hercules, USA) overnight at 30 V at 4 °C, following the Bio-Rad manual. The immunoblotting assay was conducted incubating a PVDF membrane with the 6× His tag antibodies (HRP) (GeneTex, Irvine, USA), final concentration 0.1 μg/mL.

### GUS histochemical assay

The histochemical detection assay was performed for several types of tissue in the given experiment. First, to evaluate the GUS protein activity in the swollen hypocotyl parts processed via the *R. radiobacter*-mediated transformation. Also, the histochemical detection assay was conducted after the *R. rhizogenes*-mediated transformation: preliminary on hairy root clones and on leaf fragments from the regenerated transgenic plants.

All the explants: hypocotyls, hairy roots and leaf discs were subjects to the GUS assay as recommended by Jefferson et al. (1987). The GUS assay with the X-Gluc substrate was performed with 1 mM substrate in 50 mM NaH_2_PO_4_, 0.05% v/v Triton X-100, pH 7.0 at 37 °C for 1 h. After staining, the examined fragments were incubated in 70% ethanol.

## Results

### Outcomes of the screening prephase

The tissue culture development of the selected cultivars of *B. oleracea* var. *botrytis* (Di Sicilia Violetto, Pionier, Poranek, Rober, Snowball X) was recorded after 5 weeks on the shoot regenerating medium. The Pionier cultivar seems to be the most suited for genetic engineering, providing the largest number of shoots (among all the tested cultivars—*data not shown*), mostly via the indirect pathway of regeneration. The results and characteristic of the in vitro response of the Pionier explants are presented in Additional file 1: Tables S1 and S2.

In summary, hypocotyls display robust regeneration potential. Medium *E* stimulates the strongest response considering the amount of induced callus and the number of induced and regenerated shoots.

At the same time, cotyledons display no or poor potential for regeneration (only via indirect organogenesis, with medium *C* stimulating the strongest in vitro regeneration response), rudimentary induction of the callus compared to the response of the hypocotyl. After 6 weeks, none of the cotyledons survived (observation common to all cultivars).

It was shown that among five tested media, the Pionier hypocotyls demonstrated the greatest regeneration potential when bred on medium *E*.

Since the Pionier cultivar presented the strongest in vitro response, this is also the cultivar we report the results of rooting induction for: ***A***^***r***^—87%, ***B***^***r***^—100%, ***C***^***r***^—100%, ***D***^***r***^—100%. All other tested cultivars also displayed nearly 100% of rooting (*detailed data not shown*) on all the rooting media regardless of the NAA concentration. However, the higher the NAA concentration, the more shoot (wrinkling, lancet-like leaves) and root (hairy root-like) morphology changed. Thus, the MS medium seems to be more suited for rooting stimulation, although the root formation efficiency was slightly lower.

In consequence of the screening prephase Pionier was chosen as a target cultivar for genetic transformation.

### Outcomes of the transformation

All data presented below are derived from experiments with 100 explants per treatment.

#### Outcomes of the *Rhizobium radiobacter*-mediated transformation

In general, there was no regeneration of whole plants from hypocotyls after the *R. radiobacter*-mediated transformation. Most of 100 explants transformed with EHA 105 and 100 transformed with LBA 4404 strain displayed only the initial stages of callus formation in the wounding sites of the explants; than the process of its formation stopped and the callus began to decompose. Mostly, the hypocotyls presented characteristic changes in their shape, like swelling of the ends. This phenomenon was slightly stronger in the case of the EHA 105 treated hypocotyls. Since the hypocotyls survived on the selective media I° and II°, they were considered as the potentially transgenic. Since no bacteria should be present at this stage of experiment giving false histochemical response, the *gusPlus* expression was investigated in the hypocotyl sections. However, the GUS assay hardly revealed any marks of genetically engineered tissues in the LBA treated explants and only trace marks (slight spots) mostly in the EHA treated hypocotyls. Among EHA 105 and LBA 4404-treated hypocotyl explants only 11% and 5%, respectively, showed organogenic shoot development (Additional file 1: Fig. S2). Interestingly, on the contrary to the prephase report, only direct organogenesis was observed. After 2-month evaluation of the shoot development the shoots were transferred to the rooting medium but no rooting was observed and the shoots decayed. Numerical results of the *R. radiobacter*-mediated transformation approach is presented in Table 1.

**Table 1:** Comparison of the transformation results for Pionier cauliflower cultivar

*Rhizobium* species	*R. radiobacter*		*R. rhizogenes*	
Strain	EHA 105	LBA 4404	A4	ATCC 15834
Transformation efficiency	11 % of hypocotyls with at least 1 shoot	9 % of hypocotyls with at least 1 shoot	72 % of seedlings with at least 1 hairy root	9 % of seedlings with at least 1 hairy root
% of transgenic explants confirmed in GUS assay	8^a^ % of transgenic hypocotyls confirmed in GUS assay	2^a^ % of transgenic hypocotyls confirmed in GUS assay	55 % of transgenic hairy roots confirmed in GUS assay	15 % of transgenic hairy roots confirmed in GUS assay
Regeneration efficiency	0.28 Average number of shoots per hypocotyl	0.09 Average number of shoots per hypocotyl	2.1 Average number of buds per callus	0.5 Average number of buds per callus
Rooting efficiency of transgenic shoots	0 Average number of rooted shoots per their total number	0 Average number of rooted shoots per their total number	90 Average number of rooted shoots per their total number	78 Average number of rooted shoots per their total number

^a^Trace staining (spots) at the end of hypocotyls

#### Outcomes of the *Rhizobium rhizogenes*-mediated transformation

**Fig. 1:**
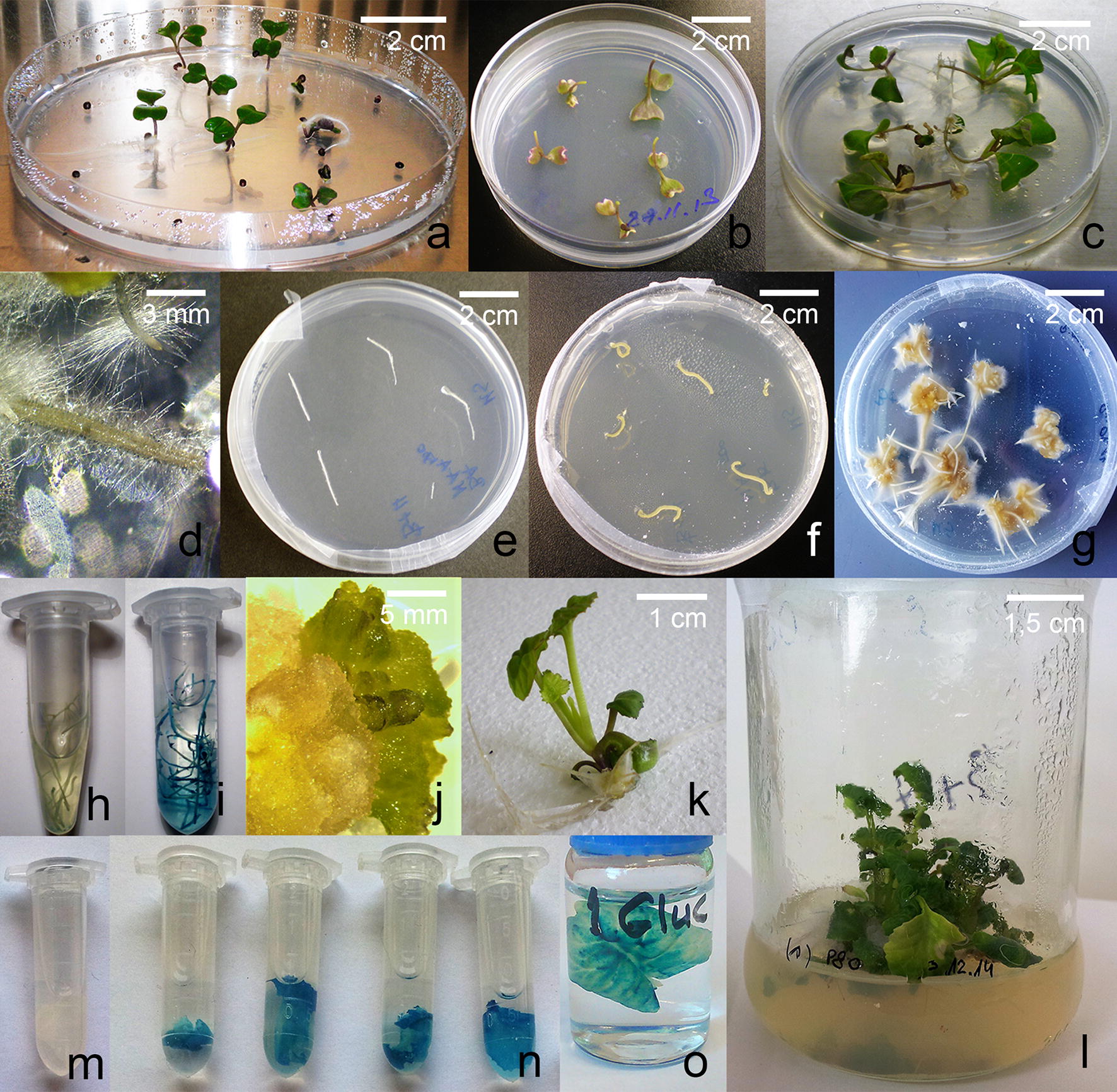
The process of *R. rhizogenes*-mediated transformation, regeneration and analysis of the *B. oleracea* var. *botrytis* (*Pionier* cultivar) plants (scheme for the A4 strain). **a** Cauliflower seed germination; **b** hypocotyls of the derooted seedlings after bacteria dropping; **c** hairy root induction on the hypocotyls; **d** an individual hairy root; **e**–**g** stages of the transgenic hairy root multiplication; **h**, **i** the GUS assay on the hairy root clones, wild type negative control and the tested probe respectively; **j** indirect organogenesis on the transgenic callus obtained on the hairy root cultures; **k** shoot development; **l** rooting of the transgenic cauliflower shoots; **m**–**o** histochemical analysis of the cauliflower leaves, wild type negative control and the tested probes respectively

Derooted seedlings were subjected to the *R. rhizogenes*-mediated transformation. 100 hypocotyl sections were treated with the ATCC 15834 or with A4 strain, yielding respectively 9% and 72% of transformation efficiency, manifesting itself as the hairy root growth. After the propagation of hairy roots, the preliminary histochemical assay was carried out. Again, since no bacteria should be present at this stage of the experiment giving false histochemical response, the *gusPlus* expression was investigated in the hypocotyl sections. The GUS assay revealed strongly genetically engineered tissues via blue tint response: 55% of confirmed transgenic hairy roots in the GUS assay for the A4 strain (Fig. 1h, i) and 15% for ATCC 15834. Next, the callus was formed on the cut edges of hairy-root explants (the scheme of development of transgenic plants is documented in Fig. 1). Callus tissue gave rise to shoot buds at a ratio of up to 0.5 and 2.1 of buds per callus. All numerical results of the *R. rhizogenes*-mediated transformation approach is presented below (Table 1). Excised adventitious shoots were rooted at a frequency of 78% and 90% within 2 weeks. Transgenic *B. oleracea* var. *botrytis* plants were successfully cultivated on the agar-solidified Murashige and Skoog medium supplemented with 20 μg/mL hygromycin and 300 μg/mL cefotaxime. Once fully regenerated plants were achieved and successfully maintained, the final histochemical assessment revealed 100% positive response for the GUS assay in cauliflower leaves. The leaves derived from the wild type plants did not show the presence and activity of transgenic glucuronidase; the leaf received from the transgenic plant was brightly blue coloured. The results confirmed correct production of the active protein by the transgenic plants.

**Fig. 2:**
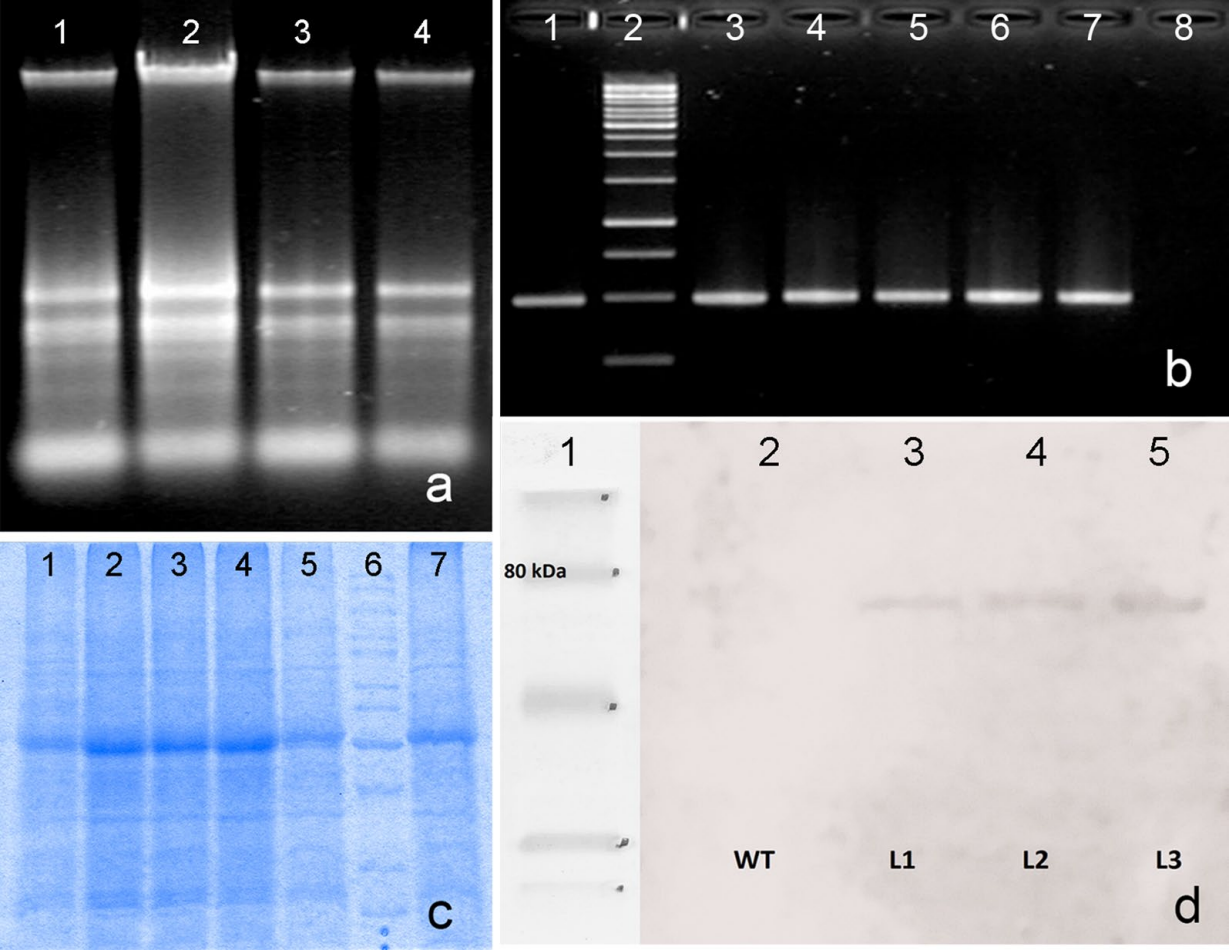
Molecular analysis of transformants. **a** Electrophoresis of genomic DNA: *lane 1*—a wild type plant (WT), *lanes 2–4*—independent transgenic lines (TL1, TL2, TL3); **b** detection of the coding sequence of the *gusPlus* gene (488 bp) in *B. oleracea* var. *botrytis* transgenic and wild-type plants. *Lane 1*—a positive control (PC, plasmid 1305.2), *lane 2*—a molecular weight marker (GeneRuler 1 kb DNA Ladder), *lanes 3–7*—independent transgenic lines (TL1, TL2, TL3, TL4, TL5), *lane 8*—a wild-type plant (WT); **c** SDS-PAGE separation of proteins from the transgenic cauliflower plants. *Lane 1*–*5*—independent transgenic lines (TL1, TL2, TL3, TL4, TL5), *lane 6*—molecular weight marker (M—GPB 260 kDa Protein Marker), *lane 7*—a wild type plant (WT); **d** detection of the *β*-glucuronidase protein in the total extractable protein isolates derived both from *B. oleracea* var. *botrytis* wild type and transgenic plants. *Lane 1*—molecular weight marker (M—GPB 260 kDa Protein Marker), *lane 2*—a wild type plant protein isolate (WT), *lanes 3,4*—independent transgenic lines (L1, L2), *lane 5*—positive control (L3). Contrary to the wild type, in the case of transgenic plant extracts a positive signal was observed (about 80 kDa)

Next, randomly chosen selective transformants, previously investigated via histochemical test were analyzed at the genomic and proteomic level. The genomic DNA isolated both from potentially transgenic and wild-type plants was used as a template for the PCR amplifications (Fig. 2a, b). The PCR procedure confirmed the presence of products with the specified length corresponding to expectations (488 bp). The isolate derived from the wild type plant did not exhibit the presence of the *gusPlus* gene.

To investigate the transgenicity of the proteome, the total extractable proteins were isolated from randomly selected leaf tissues. The SDS-PAGE analysis showed the presence of separated protein bands. Bands corresponding to the molecular mass of *β*-glucuronidase fused to the ELP tag (~ 70 kDa) were not revealed in the electrophoregrams (Fig. 2c).

Finally, the western-blot analysis of transgenic cauliflower plants yielded one main protein fraction corresponding to the expected protein product size (~ 70 kDa), not observed in the wild-type control extract (Fig. 2d). Although the signal was weak, there was a significant and visible difference on the membrane between the two types of extracts. That is a direct confirmation of the presence of β-glucuronidase-ELP fusion. Direct confirmation of the presence of an active protein—the histochemical assessment—was already presented.

## Discussion

The production of transgenic plants in cauliflower is considered difficult. As it is known from biotechnological research data, it is not the only species that displays troublesome recalcitrance towards genetic manipulations (e.g. *Phaseolus vulgaris*; *Brassica napus*) (Hnatuszko-Konka et al. 2014; Liu et al. 2015). As in the case of other important crops, the establishment of a reliable procedure of transformation would provide the space for their improvement or biopharming. Therefore, we presented a simple procedure for the *R. rhizogenes*-mediated transformation via the indirect regeneration process in cauliflower hypocotyl explants. The reported procedure is an outcome of a holistic analysis started from the evaluation of the regeneration potential of cauliflower cultivars and the ability of media to trigger it.

### Screening prephase—regeneration

The results of the prephase let us select the most promising cultivar—Pionier—in terms of tissue culture response, although we are aware of the limited number of cultivars screened. On the other hand, it is generally agreed today that cauliflower conveniently does not require any specific treatment during in vitro maintenance and the research on its tissue cultures yielded quite repeatable and effective protocols. Previous protocols displayed high regeneration efficiency in *B. oleracea* var. *botrytis* plants for many types of explants (Kieffer and Fuller 2013; Leroy et al. 2000). Pavlović and colleagues (2010) precisely indicated that the percentage of in vitro responding explants critically depended on the explant type, and they pointed out hypocotyls as the best starting material for the majority of the cole crops. However, it was cauliflower that exceptionally displayed the highest regeneration potential when regenerated from cotyledonary, not hypocotyl, explants (Pavlović et al. 2010). A similar observation about cotyledon domination was reported by Bhalla and Smith (1998). That was the reason why we chose both cotyledons and hypocotyls. However, our research revealed the opposite outcome: cotyledons displayed no or poor regeneration and rudimentary induction of callus compared to the response of hypocotyls which displayed robust regeneration potential. Thus, our results were consistent with the literature data that mostly suggest that the hypocotyl of cauliflower seems to be an explant of choice for the studies on regeneration, pointing at its amenability to in vitro handling (Brown and Wang 2004; Lingling et al. 2005; Smith and Bhalla 1998). The reported incompatibility can be explained by different media used and substantial differences in the response to regeneration resulting from genomic influence (Vinterhalter et al. 2007).

Next, the tissue culture potential of the aforementioned explants was tested on five medium variants based on the standard MS medium with addition of 6-benzylaminopurine and 1-naphthaleneacetic acid. Growth hormones were used alone (BAP) or in conjunction to stimulate regeneration. In the case of cotyledons only indirect organogenesis was observed on two compound variants (BAP and NAA), although this does not represent statistically different results (*data not shown*). It was also only the combinations of phytohormones that yielded actual callus formation which, on the side note, was slow in culture. In comparison, the induced hypocotyl explants brought shoots via both indirect and direct organogenesis. The percentage pattern of both, the particular pathways and shoots and/or roots induced, were changed as different concentrations of the particular phytohormones were provided. With the increase in BAP concentration the shoot formation (understood as the total number of shoots without pathway distinction) grew gradually, confirming the rule that a high ratio of cytokinin-to-auxin induced shoot regeneration (Ikeuchi et al. 2013; Liu et al. 2015). On the contrary, the cauliflower callus seemed to be induced (in hypocotyls of the Pionier cultivar) only in the presence of both regulators and at a high ratio of BAP to NAA, which is not a typically observed relation (it usually takes an equal cytokinin-to-auxin ratio) (Ikeuchi et al. 2013). In the research conducted by Kumar Ray and colleagues (2012), BAP containing shoot induction medium appeared to be sufficient to get callus formation. Again, the influence of the group and concentration of growth regulators on the tissue culture of cauliflower varied with the genotype (here cultivar) tested. In general, also in our experiments the cytokinin (BAP) was the key phytohormone group inducing bud organogenesis from *B. oleracea* var. *botrytis*, however the optimal concentration appeared cultivar-specific.

As it was mentioned, the relation of direct and indirect organogenesis changed depending on the cytokinin-to-auxin ratio. In summary, with increasing BAP concentration the contribution of indirect organogenesis in general regeneration response increased. However, it seems that indirect/direct organogenesis ratio in hypocotyls slightly decreases when BAP/NAA was around the middle values e.g. BAP/NAA ≈ 10. While a less or more extreme cytokinin-to-auxin relation was used (like BAP/NAA ≈ 5 or 20), intermediate callus appeared. However, it is difficult to confront such an observation since it was made for a particular concentration of PGRs (plant growth regulators) and a particular cultivar.

Developed from the callus, adventitious buds and subsequently shoots elongated, were left for rooting on root inducing media. Similarly to other literature data (Baskar et al. 2016; Kumar Ray et al. 2012; Pavlović et al. 2010), we found the basal MS medium sufficient to stimulate rooting. The addition of NAA progressively increased the effectiveness of root formation, but seemed to cause alteration of morphology of both roots and whole shoots. Similar influence was reported by Gerszberg et al. (2015) in *B. oleracea* var. *capitata*.

### Transformation

The key goal of the conducted experiment was to establish a procedure of genetic engineering that could be used to enter specific traits to the existing varieties of cauliflower. The general idea of the *Rhizobium*-mediated transformation of the *B. oleracea* var. *botrytis* plants originated from its domination over other transformation approaches (Kong et al. 2009). Here, the use of both *R. radiobacter* and *R. rhizogenes* vectors resulted from the poor outcome of the transformation mediated by the first vector. At first, the experiment was meant to adapt only the protocol developed by Chakrabarty and colleagues (2002) to evaluate the virulence potential of LBA 4404 and EHA 105. We then followed Chakrabarty’s suggestion recommending conditions for subsequent experiments, which however did not prove correct in the case of the tested bacterial strains. The poor transformation efficiency (5–11%) obtained for this kind of agroinfection could have resulted from several reasons. First of all, the protocol may just not work for the Pionier cultivar, indicating again the often reported genotype limitations (Chakrabarty et al. 2002; Kumar Ray et al. 2012). Secondly, the protocol we based our experiment on was recommended for the GV2260 strain. Still, our results were worse even when compared to Chakrabarty’s outcomes with LBA 4404 and EHA 105 (the effect of cultivar?). Finally, there is a question of the amenability of explants to genetic manipulations. Kumar Ray et al. (2012) pointed out that although hypocotyls might produce more shoots than cotyledons, it was still possible that cotyledons presented a better target for the transformation of cauliflower. Similar observation about cotyledon domination in terms of genetic modifications was reported by Bhalla and Smith (1998). If that is the case, it would mean we need to focus again on in vitro culture of cotyledons.

Since the chosen cultivar of *B. oleracea* var. *botrytis* appeared to be resistant even to quite hypervirulent *R. radiobacter* strains (although we are aware that the protocol may just need additional optimization for this cultivar), a completely different approach was considered.

To the best our knowledge there is a relatively limited amount of protocols for transformation of cauliflower and the *R. rhizogenes*-mediated transformation usually gives way to the *R. radiobacter* vector. Therefore, we decided to test the potential of *R. rhizogenes.* Whole transgenic plants were regenerated from cauliflower hairy-root cultures via intermediate callus. Two *R. rhizogenes* strains—ATCC 15834 and A4—were tested while evaluating the viability of this transformation method for cauliflower improvement, unambiguously pointing to the A4 strain as the more effective one. Whether the protocol applied is genotype-independent has to be proved by further research. However it turned out to be strain dependent. It assured high transformation efficiency (72%) only when the A4 *R. rhizogenes* strain was used (only 9% for ATCC 15834).

It should be pointed out that our transformation efficiency (55% determined by the GUS assay) outruns not only previous results obtained by *R. rhizogenes*, but also efficiencies obtained using *R. radiobacter*. Chakrabarty et al. (2002) reported maximum transformation frequency (evidenced by GUS staining) of 22.6%. A similar range of transformation efficiencies was reported by Yu and co-workers in 2010 (21.6% and 18.3% for cotyledons and hypocotyls). In such context our protocol assures at least 2.5 fold increase in frequency of genetic modification (and more than sixfold increase when compared only to *R. rhizogenes*-mediated transformation).

A similar protocol utilizing *R. rhizogenes* (A4T) was reported by Puddephat et al. (2001). It was successfully applied to produce transgenic broccoli and cauliflower. The most important differences were as follows: the optical density used (≥ 1.0 in comparison to ≈ 0.3 in our studies), the way of inoculation (dropping versus immersion in our studies), and the age of seedlings undergoing transformation (4-day-old aseptic seedlings in comparison to 10-day-old in our studies). Our parameters yielded the transformation efficiency of 72% (the number of seedlings with at least 1 hairy root) and of 55% (measured as the number of inoculated hypocotyls producing at least 1 transgenic hairy-root determined by the GUS assay). In comparison, Puddephat et al. (2001) obtained the maximum transformation efficiency of 8.7% for histochemically confirmed hairy-roots. Establishing the aforementioned parameters we relied on earlier studies reporting that e.g. lowering bacterial density had a beneficial influence on the transformation effectiveness (Chakrabarty et al. 2002; Henzi et al. 2000). We also believe that a combination of low optical density and optimal seedling age decreases the necrosis rate among explants (although we are aware that younger explants display greater regenerative potential; thus, a compromise has to be established between viability and regenerative rate).

Our observations were consistent with literature data (David and Tempé 1988; Puddephat et al. 2001) concerning the morphology of plants. The transgenic plants exhibited an altered phenotype, although it varied between individuals from the same transformation event. It must be pointed out, however, that Puddephat et al. (2001) noticed such alteration only in transgenic cauliflower, we observed a certain degree of dwarfism, leaf wrinkling or lancet-like leaves and hairy root-like rooting also in wild-type plants rooted on a high concentration of NAA. It seems that the phenotype of newly regenerated transgenic plants after the *R. rhizogenes* mediated transformation changed in a way similar to that caused by a high dose of auxin (or maybe just NAA). Whether such altered morphology is preserved in progeny is still to be investigated (Puddephat and co-workers observed normal phenotype in progeny). The possible relation between phenotype, content of opines, T-DNA content and/or expression was discussed in 1988 by David and Tempé who reported a modified phenotype in regenerated transformants.

The transgenic character of the obtained cauliflower was unequivocally confirmed by various methods and at different levels, from selective breeding through genome and proteome analysis. Nonetheless, an interesting question remains about the reason for the negative SDS-PAGE and for the undeniably rudimentary response to the immunochemical reaction. The immunoblotting analysis revealed a protein fraction of the expected mass, not observed in the wild-type extract. It may suggest a low protein content in the investigated extracts, which in turn may be caused by a low protein concentration in the plant tissues or in the extract itself due to the imperfect extraction procedure. The former may result from e.g. silencing etc., but it would be in certain conflict with the fast histochemical reaction observed. Therefore it is more likely that the heterologous protein is synthesised and gains its mature biological activity, and the given state of affairs may result from the extraction difficulties. Similar issues are widely described in the literature (Hnatuszko-Konka et al. 2016). In addition, as it was tested by Barański and Puddephat (2004), in the cauliflower leaves glucuronidase expressions from the extensin promoter is among the lowest when compared to other organs. Very much has also been written and said about the optimization of the extraction conditions that was tested for a great number of proteins on a case-by-case basis. Many authors mention the development of plant species-dependent approaches (Wilken and Nikolov 2012). However, whether this optimization of the protocols of proteome isolation is sufficient also for the cauliflower tissues is still to be verified.

In conclusion, a simple and reproducible procedure for *R. rhizogenes*-mediated transformation of hypocotyl sections (of derooted seedlings) of 10-day-old seedlings of cauliflower has been developed.

## Additional file

**Additional file 1: Fig. S1.** A circular plasmid map of the modified pCAMBIA 1305.2 vector. **Fig. S2.** The regeneration from the Pionier cauliflower hypocotyls after *R. radiobacter*-mediated transformation approach: left side (a, c, e, g) after EHA 105 treatment, right side (b, d, f, h) after LBA 4404 treatment. a, b – growth of the hypocotyls on the selection medium I°; c, d – 10-day growth of the hypocotyls on the selection medium II° (shoot induction); e, f – 2-month shoot regeneration on the selection medium II° (all the regenerated shoots are gathered on one Petri dish per given strain); g, h – the effects of the GUS assay on the Pionier cauliflower hypocotyls after *R. radiobacter*-mediated transformation approach (EHA 105 and LBA 4404 strain respectively) .**Table S1.** The characteristic of the in vitro response of the Pionier hypocotyl explants to different variants of regeneration media. **Table S2.** The characteristics of the in vitro response of the Pionier cotyledon explants to different variants of regeneration media.
