# Supplementary material for: High efficiency transformation of Brassica oleracea var. botrytis plants by Rhizobium rhizogenes
Source: AMB Express. 2018 Aug 6;8:125. doi: 10.1186/s13568-018-0656-6 (PMC6077290; doi:10.1186/s13568-018-0656-6)
Supplement: Supplementary file 1 — Additional file 1: Fig. S1. A circular plasmid map of the modified pCAMBIA 1305.2 vector. Fig. S2. The regeneration from the Pionier cauliflower hypocotyls after R. radiobacter-mediated transformation approach: left side (a, c, e, g) after EHA 105 treatment, right side (b, d, f, h) after LBA 4404 treatment. a, b – growth of the hypocotyls on the selection medium I°; c, d – 10-day growth of the hypocotyls on the selection medium II° (shoot induction); e, f – 2-month shoot regeneration on the selection medium II° (all the regenerated shoots are gathered on one Petri dish per given strain); g, h – the effects of the GUS assay on the Pionier cauliflower hypocotyls after R. radiobacter-mediated transformation approach (EHA 105 and LBA 4404 strain respectively) .Table S1. The characteristic of the in vitro response of the Pionier hypocotyl explants to different variants of regeneration media. Table S2. The characteristics of the in vitro response of the Pionier cotyledon explants to different variants of regeneration media. [file 13568_2018_656_MOESM1_ESM.pdf]

Additional file 1

AMB Express

Title: **High efficiency transformation of *Brassica oleracea* var. *botrytis* plants by *Rhizobium rhizogenes***

Tomasz Kowalczyk *****, Aneta Gerszberg, Paulina Durańska, Róża Biłas, Katarzyna Hnatuszko-Konka*****

***** These authors contributed equally to this article (co-first authorship)

Department of Genetics, Plant Molecular Biology and Biotechnology

Faculty of Biology and Environmental Protection,

University of Lodz; 90-237 Lodz, Banacha 12/16, Poland

1. **Tomasz Kowalczyk** e-mail – tomasz.kowalczyk@biol.uni.lodz.pl
2. **Aneta Gerszberg** – e-mail aneta.gerszberg@biol.uni.lodz.pl
3. **Paulina Durańska** – e-mail p.duranska@wp.pl
4. **Róża Biłas** – e-mail bilasshylo.r@gmail.com
5. **Katarzyna Hnatuszko-Konka** ORCID: 0000-0001-8174-3268 – corresponding author: e-mail katarzyna.hnatuszko@biol.uni.lodz.pl phone +48 42 635 42 19, mobile +48 692 434 221, fax + 48 42 635 44 23.

Legends to figures:

Additional file 1: Fig. S1 A circular plasmid map of the modified pCAMBIA 1305.2 vector

Additional file 1: Fig. S2 The regeneration from the Pionier cauliflower hypocotyls after *R. radiobacter*-mediated transformation approach: left side (a, c, e, g) after EHA 105 treatment, right side (b, d, f, h) after LBA 4404 treatment. a, b – growth of the hypocotyls on the selection medium I°; c, d – 10-day growth of the hypocotyls on the selection medium II° (shoot induction); e, f – 2-month shoot regeneration on the selection medium II° (all the regenerated shoots are gathered on one Petri dish per given strain); g, h – the effects of the GUS assay on the Pionier cauliflower hypocotyls after *R. radiobacter*-mediated transformation approach (EHA 105 and LBA 4404 strain respectively)

Additional file 1: Fig. S1

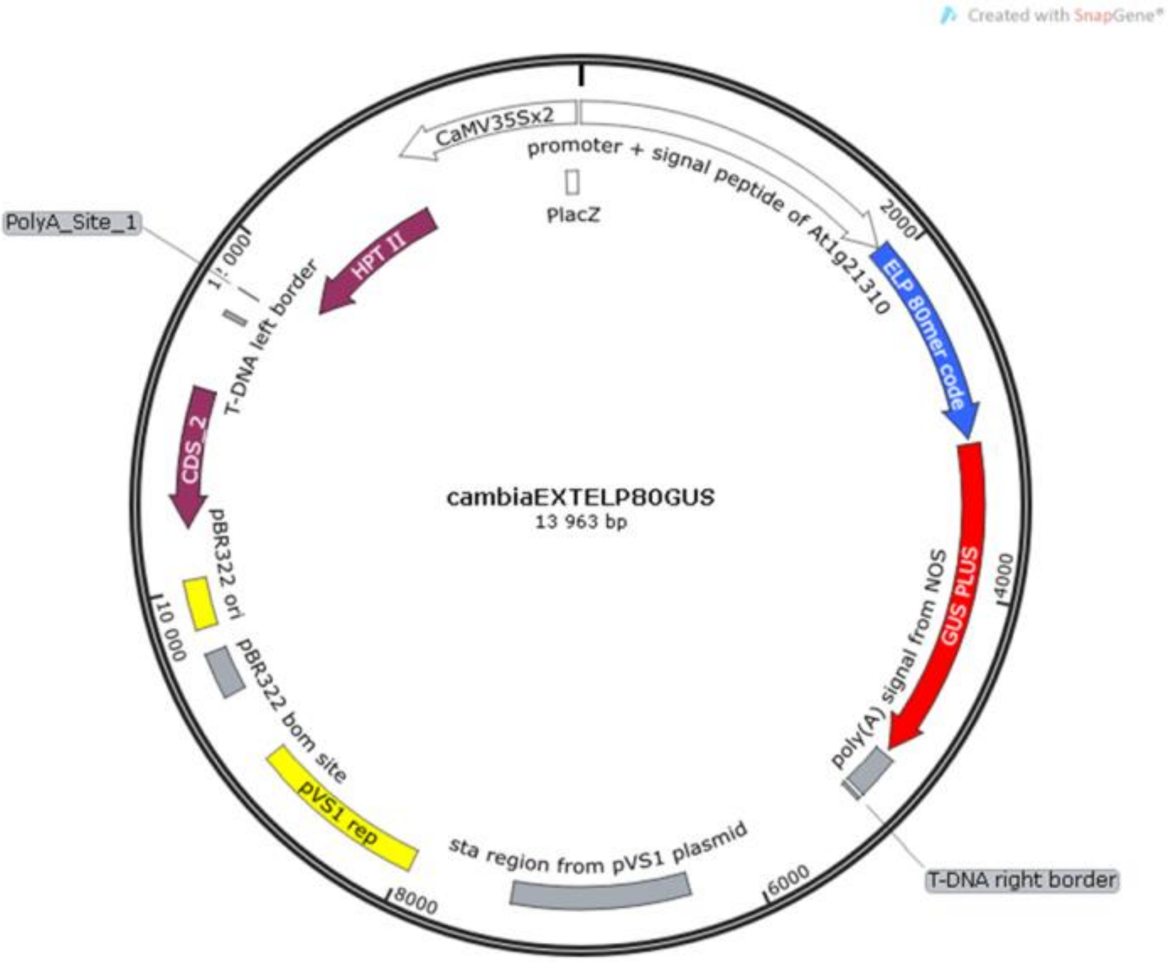

Additional file 1: Fig. S2

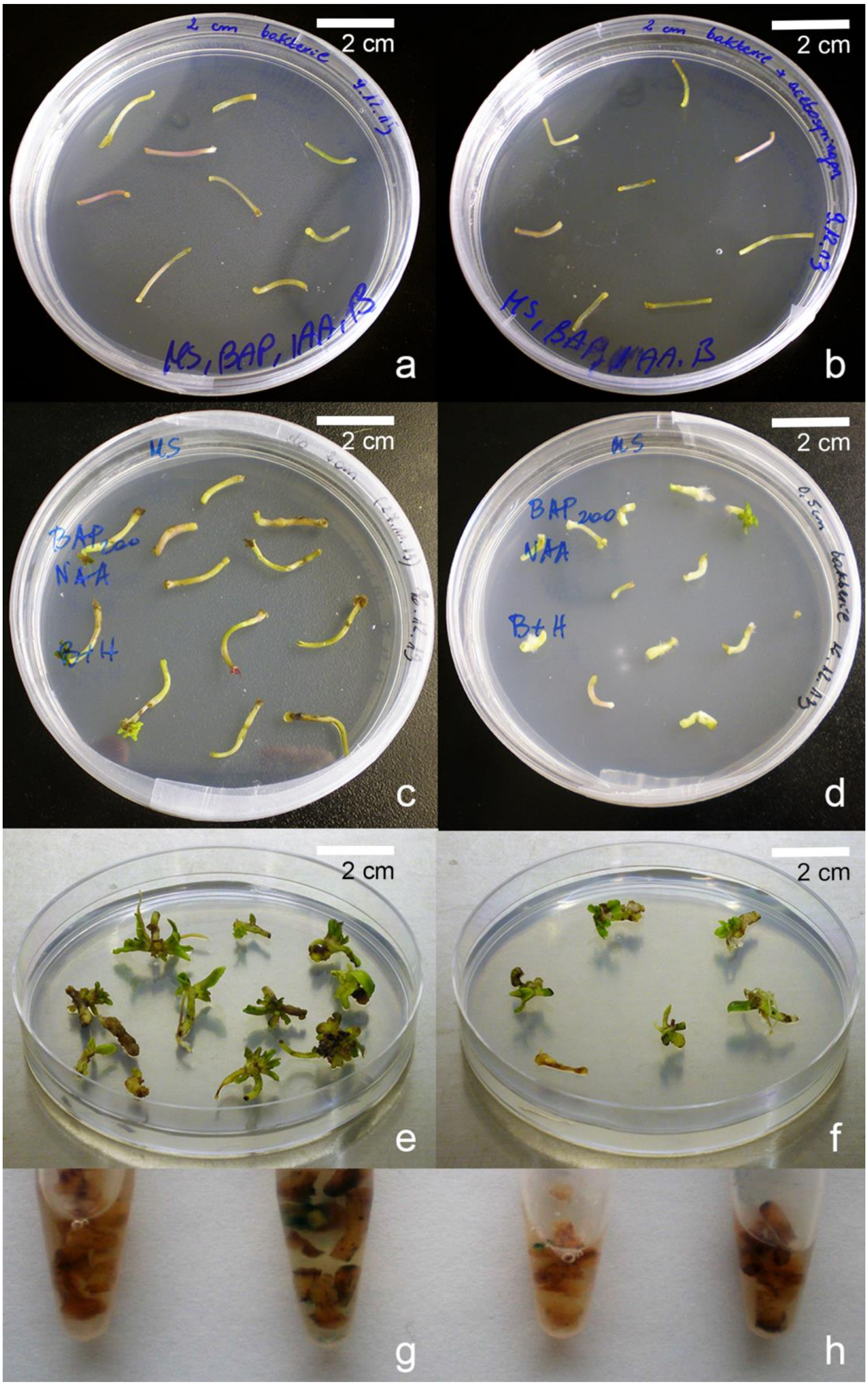

Additional file 1: Table S1 The characteristic of the in vitro response of the Pionier hypocotyl explants to different variants of regeneration media

**Medium/ image no.**	**Observation, conclusion**	**No. of explants with callus induction (%)**	**No. of explants with shoots (%)**	
			**Callus intermediate**	**Direct organogenesis**
A	The strongest stimulation of the rooting, no induction of the callus, no swelling at the ends of explants; rooting process observed (++)	0	62	
			0	100
B	Swelling at the ends of explants, but relatively slight induction of the callus, poor induction and regeneration of the shoots; no rooting (-)	0	50	
			0	100
C	C, D, E media cause similar response, stronger swelling at the ends of explants, strong induction of the callus, the highest rate of the induction and regeneration of the shoots, some rooting events; rooting (+)	68	67	
			66.67	33.33
D		70	70	
			57.14	42.86
E		90	80	
			75	25

Additional file 1: Table S2 The characteristics of the in vitro response of the Pionier cotyledon explants to different variants of regeneration media

**Medium/ image no.**	**Observation, conclusion**	**No. of explants with callus induction (%)**	**No. of explants with shoots (%)**	
			**Callus intermediate**	**Direct organogenesis**
A	No or slight cotyledon growth, no induction of the callus, fast necrotic response; no rooting	0	0	
			0	0
B	Slower necrotic process, no or slight cotyledon growth, occasional induction of the callus; no rooting	80 (only initiation level)	0	
			0	0
C	Induction of the callus, occasional rooting events, the highest cotyledon growth rate;	82	18	
			100	0
D	Occasional induction of the callus, rooting process observed more often, medium cotyledon growth rate;	7	0	
			0	0
E	The strongest induction of the callus growth, a single induction of the shoot and direct regeneration event, however, after poor rooting and growth inhibition, the necrotic process occurs;	93	14	
			100	0
